# Acupuncture of polycystic ovary syndrome: delving into bile acid metabolism

**DOI:** 10.1186/s13020-025-01297-6

**Published:** 2026-01-09

**Authors:** Haolin Zhang, Yang Chen, Dai Heng, Jingzhi Luo, Haining Wang, Wei Wang, Zejun Huo, Rui Wei, Yang Ye, Xiaoyu Long, Chunmei Zhang, Wen Ma, Li Shi, Yang Yang, Chunhua Shan, Shuhan Yang, Rong Li, Dong Li, Jie Qiao

**Affiliations:** 1 State Key Laboratory of Female Fertility Promotion, Center for Reproductive Medicine, Department of Obstetrics and Gynecology, Peking University Third Hospital, Beijing, 100191 China; 2 Department of Traditional Chinese Medicine, Peking University Third Hospital, Beijing, 100191 China; 3 Department of Integration of Chinese and Western Medicine, Peking University Health Science Center, Beijing, 100191 China; 4 Center for Precision Medicine Multi-Omics Research, Institute of Advanced Clinical Medicine, Peking University, Beijing, 100191 China; 5 Beijing Advanced Center of Cellular Homeostasis and Aging-Related Diseases, Institute of Advanced Clinical Medicine, Peking University, Beijing, 100191 China; 6 Department of Endocrinology and Metabolism, Peking University Third Hospital, Beijing, 100191 China; 7 Department of Obstetrics and Gynecology, Peking University Third Hospital, Beijing, 100191 China; 8 Department of Endocrinology and Metabolism, State Key Laboratory of Female Fertility Promotion, Peking University Third Hospital, Beijing, China; 9 NHC Key Laboratory of Cardiovascular Molecular Biology and Regulatory Peptides, Peking University, Beijing, China; 10 State Key Laboratory of Natural and Biomimetic Drugs, School of Pharmaceutical Sciences, Peking University, Beijing, 100191 China; 11 National Clinical Research Center for Obstetrics and Gynecology (Peking University Third Hospital), Beijing, 100191 China; 12 Ministry of Education, Key Laboratory of Assisted Reproduction (Peking University), Beijing, 100191 China; 13 Beijing Key Laboratory of Reproductive Endocrinology and Assisted Reproductive Technology, Beijing, 100191 China

**Keywords:** Polycystic ovary syndrome, Acupuncture, Bile acids, Insulin resistance, FXR pathway

## Abstract

**Background:**

Polycystic ovary syndrome (PCOS), a prevalent endocrine disorder, is characterized by hyperandrogenism, ovulatory dysfunction, and polycystic ovarian morphology. In light of the critical role of bile acids in metabolic regulation and the therapeutic potential of acupuncture for endocrine–metabolic disorders, this study aims to explore the effects of acupuncture on bile acid metabolism and insulin sensitivity in both PCOS patients and rat models.

**Methods:**

33 PCOS participants and 28 age/BMI-matched controls were enrolled in a clinical trial (NCT04193371). PCOS participants received 4 month acupuncture plus lifestyle (A&L) or a sham acupuncture plus lifestyle (SA&L) intervention followed by 4 month observation. Serum bile acids were profiled by LC–MS/MS. Multiple hormones and inflammatory markers were analyzed by enzyme-linked immunosorbent assay and insulin sensitivity was evaluated through oral glucose tolerance test and insulin tolerance test. A dihydrotestosterone -induced PCOS rat model was established and treated with acupuncture. The estrous cycle and ovarian morphology were assessed using HE staining, insulin resistance was evaluated and hormone levels were measured. Transcriptome profiling of hepatic tissues was conducted in a PCOS rat model to delineate molecular alterations associated with acupuncture intervention, with particular emphasis on genes involved in bile acid biosynthesis.

**Results:**

The homeostasis assessment of insulin resistance (HbA1c, HOMA, AUC) of PCOS is significantly reduced after acupuncture compared to the pre-intervention (p < 0.05). Surprisingly, simultaneously ameliorated outcomes included the body mass index (BMI), sex hormone-binding globulin (SHBG), free androgen index (FAI) and anti-Müllerian hormone (AMH). The potency lasted for another 4 months, indicating the enduring effects of the acupuncture regimen. Metabolic improvements were associated with changes in specific bile acids (e.g., taurocholic acid, lithocholic acid). In PCOS rat models, acupuncture restored regular estrous cycles, reduced the incidence of ovarian cysts and improved the insulin resistant. Transcriptomic analysis of rat liver revealed that acupuncture significantly reversed the expression of genes associated with bile acid metabolism and the FXR signaling pathway.

**Conclusions:**

Acupuncture therapy offers potential therapeutic benefits to PCOS women, with mechanisms involving the bile acid–FXR axis potentially contributing to improvements in insulin resistance and other disease-related symptoms.

## Background

Polycystic Ovary Syndrome (PCOS), the most prevalent endocrine-metabolic disorder in women of reproductive age, is characterized by menstrual irregularities, hyperandrogenism, and anovulation. Its incidence, particularly among adolescents, has risen significantly in recent decades [1]. Current medical interventions for PCOS primarily focus on symptom managements, however, it is important to note that the effectiveness of these practices is frequently unsatisfactory but accompanied by many side effectst [2]. Acupuncture, one of the most ancient medical therapies and as a crucial segment of traditional Chinese medicine has garnered global research interest due to its unique mechanisms and favorable safety profile. A couple of studies indicate that acupuncture shows potential in ameliorating endocrine-metabolic dysregulation in PCOS [3, 4]. Previous studies, including our own, have established its therapeutic efficacy across disease stages, particularly in mitigating insulin resistance [5]. Nevertheless, the underlying molecular mechanisms remain incompletely understood.

Insulin resistance (IR) is a central pathophysiological mechanism underlying both metabolic dysfunction and reproductive impairment in PCOS and represents a pivotal therapeutic target [6]. Recent studies indicate that disruptions in the gut microbiota–bile acid axis in PCOS exacerbate hyperinsulinemia and hyperandrogenism, thereby impairing ovulatory function [7]. Our prior work revealed that PCOS is associated with altered bile acid metabolism, characterized by markedly reduced levels of glycodeoxycholic acid (GDCA) and tauroursodeoxycholic acid (TUDCA) [8, 9]. These metabolic changes correlate with immune dysregulation, compromised intestinal barrier integrity, and hyperandrogenemia [9, 10]. Alterations in bile acid composition may perturb critical signaling pathways, notably the farnesoid X receptor (FXR)-FGF15/19 axis, leading to exacerbated metabolic dysfunction including insulin resistance [9, 11]. This pathogenic cascade is further corroborated by pharmacological evidence: metformin intervention potently suppresses intestinal FXR signaling [12], mechanistically achieving through inhibition of Bacteroides fragilis-derived bile acid hydrolase activity. Consequent elevation of glycoursodeoxycholic acid (GUDCA) levels mediates FXR pathway suppression, thereby driving measurable improvements in glucose metabolism [13, 14]. Collectively, these findings highlight bile acid metabolic modulation as a pivotal therapeutic node for ameliorating insulin resistance in PCOS patients.

Acupuncture exerts microbiome-modulating therapeutic effects through selective activation of acupoints, specifically enriching beneficial taxa including Akkermansia and Prevotella [15]. This microbial remodeling activates downstream glucose metabolism pathways while concurrently suppressing proinflammatory cytokine expression. Mechanistically, the intervention upregulates both mRNA expression and protein abundance of glucose transporter GLUT4, thereby ameliorating insulin resistance through enhanced cellular glucose uptake [13]. In addition, previous studies have reported that acupuncture can ameliorate gut microbiota dysbiosis in patients with opioid use disorders (OUDs) by upregulating bile acid metabolism [16]. Notably, whether acupuncture modulates bile acid metabolism in PCOS patients—thereby exerting therapeutic effects on insulin resistance—remains unknown, with no clinical or mechanistic studies reported.

Collectively, this study explored the potential therapeutic effects of electroacupuncture on insulin resistance in PCOS through a rigorously designed randomized controlled clinical trial, complemented by mechanistic investigations in a hyperandrogenemic rat model of PCOS. Integrated clinical and animal experimental findings indicate that electroacupuncture may influence the bile acid–FXR axis, thereby contributing to the improvement of metabolic dysfunction associated with PCOS. Such results provide preliminary insights that could guide future refinement of clinical management strategies, while advancing mechanistic understanding from bench to bedside.

## Methods

### Participants

All participants provided their written informed consent before participant. The inclusion criteria for the study were age from 20 to 40 and BMI ranging from 25 to 40 kg/m^2^. Participants would be excluded if they: (1) diagnosis of any other endocrine disorder (e.g., androgen-secreting tumors, thyroid dysfunction or diabetes etc.); (2) receive pharmacological or acupuncture treatment within the last 3 months; (3) pregnancy or breastfeeding within the past 6 months.

### Sample size calculation

The sample size for each group was calculated using the following equation: 52 patients (26 in PCOS group; 26 in the control group) for LCA, 72 patients (36 in PCOS group; 36 in the control group) for TCA, 64 patients (26 in PCOS group; 26 in the control group) for TUDCA, 108 patients (54 in PCOS group; 54 in the control group) for TDCA would provide 80% power to reject the null hypothesis of equal means based on the equation below. In our study, we have analyzed 59 patients (31 in PCOS group, 28 in control group).

$${\text{n}}_{2} \,{ = }\,\frac{{\left( {{\text{z}}_{{{1} - \frac{a}{{2}}}} \,{ + }\,{\text{z}}_{1 - \beta } } \right)^{2} \left( {{\text{sd}}_{1}^{2} \,{ + }\,{\text{sd}}_{2}^{2} } \right)\left( {{1}\, + \,\frac{1}{{\text{k}}}} \right)}}{{2\left( {{\text{mean}}_{1} - {\text{mean}}_{2} } \right)^{2} }}{\text{, n}}_{1} \,{ = }\,{\text{k}}\, \times \,{\text{n}}_{2}$$

### Intervention

Eligible subjects are randomized to either acupuncture plus lifestyle (A&L) group or a sham acupuncture plus lifestyle (SA&L) intervention, the selected acupoints were determined according to findings from our previous clinical trial [17], multicenter randomized controlled trials, and a well-characterized acupuncture regimen commonly employed in PCOS animal studies [18]. Sterilized disposable needles are used, with manual stimulation to elicit de qi, and select points receive low frequency electroacupuncture. Sham acupuncture involves superficial needling at non-acupuncture points without stimulation. All participants receive structured lifestyle intervention supported by a patented digital health system through web based (Combine the website, app and wearable device) health coaching, including personalized nutrition and physical activity guidance aligned with WHO recommendations. Outcomes are assessed at baseline, after 4 months of intervention, and at 4 month follow-up. Clinical outcomes will be evaluated at baseline, 4 month after treatment and 4 month follow-up after treatment termination. The primary outcome measure is insulin resistance before and after treatment. The secondary outcomes include anthropometric measures, endocrine and metabolic outcomes.

### Outcome measurements

The primary outcome is the change of homeostatic model assessment (HOMA)-IR [fasting insulin (μU/mL) × fasting glucose (mmol/L)]/22.5)] [19]

Secondary outcome measures include other anthropometric measures, endocrine and metabolic outcomes, side-effects and adverse events.

*Anthropometric measures:* the change of BMI from baseline to the end of treatment (16th week). Body weight (kg) and body height (cm) are recorded to the nearest 0.1 kg and 0.1 cm, respectively. BMI is calculated as body weight (kg) divided by squared body height (m2). The formula for BMI is as follows: BMI = mass (kg)/(height(m))2. All women will be examined with a body composition analyzer (Tanita Corporation, model MC-180, Japan) to measure total body fat, visceral fat mass and ratio, lean mass and basal metabolic rate etc. Hirsute assessment by Ferriman-Gallway (FG), acne standard acne lesion counts, and pelvic examination will also be conducted [20].

*Metabolic measures:* insulin response to glucose during the OGTT (AUC using the trapezoidal rule) and direct analyses of fasting blood samples of insulin an glucose to enable calculation of HOMA-B/islet β-cell function [20 × fasting insulin (mU/mL)/(fasting plasma glucose (mmol/L) − 3.5)] [21]. Further, fasting blood samples are collected and saved for later analyses.

*Endocrine measures:* blood samples will be drawn for analyses of sex steroids (luteinizing hormone (LH), follicle stimulating hormone (FSH), testosterone(T), androstenedione (T2), estrogen (E2), prolactin (PRL)), by the validated gas and liquid chromatography/tandem mass spectroscopy technique, as well as sex hormone binding globulin (SHBG), Free Androgen Index (FAI), antimüllerian hormone (AMH). Fibroblast growth factor (FGF)−19, Ghrelin, Interleukin (IL)−6, beta (β)-Endorphin and adrenocorticotropic-hormone (ACTH) levels were measured by an ultrasensitive two-site enzyme-linked immunosorbent (ELISA) assay (Ansh Laboratories LLC; Webster, TX, USA). Gamma amino butyric acid (GABA) was quantified using ultra-performance liquid chromatography-tandem mass spectrometry (UPLC-MS/MS) method [7]. Tumor Necrosis Factor alpha (TNF-a) was determined by immune-assay (Luminex 100^™^, Austin, TX, US), glutamate (Glu) and dopamine (DA) plasma concentrations were measured using high-performance liquid chromatography(Phenomenex, Torrance, CA USA) [5].

### Bile acid profiling by LC–MS/MS

External bile acid standards including cholic acid (CA), α-muricholic acid (α-MCA), β-muricholic acid (β-MCA), dehydrocholic acid(DHCA), ursodeoxycholic acid (UDCA), hyodeoxycholic acid (HDCA), chenodeoxycholic acid (CDCA), deoxycholic acid(DCA), lithocholic acid (LCA), taurocholic acid(TCA), tauro-α-muricholicacid (T-αMCA), and tauro-β-muricholic acid(T-βMCA), TUDCA, taurohyodeoxycholic acid (THDCA), taurochenodeoxycholic acid (TCDCA), taurodeoxycholic acid (TDCA), taurolithocholic acid (TLCA), glycocholic acid (GCA), glycolithocholic acid(GLCA), glycoursodeoxycholicacid (GUDCA), glycochenodeoxycholic acid (GCDCA), GDCA were purchased from Sigma-Aldrich. The deuterated internal standardscholic acid-2,2,4,4-D4 (CA-d4), lithocholicacid-2,2,4,4-D4 (LCA-d4), ursodeoxycholicacid-2,2,4,4-D4 (UDCA-d4) were also obtained from Sigma-Aldrich. To conduct a quantitative analysis of bile acids, 50 µl of plasma were taken and mixed with 300 µl of ice-cold methanol and 10 µl of an internal standard mixture, followed by liquid chromatography—mass spectrometry analysis. Targeted analysis of bile acid was performed using Ekspert ultra-LC 100 coupled to Triple TOF 5600 (AB SCIEX) with a C18 BEH column (2.1*100 mm, 1.7 μm; Waters) via Multiple Reaction Monitoring in negative ionization mode. The Peak View 1.2 was used to identify bile acid and Multi Quant 2.1 was used to quantity bile acid based on the m/z value and sample retention time.

### Animals experiment

This study was conducted with the approval of the Animal Care and Use Committee at the Peking University Health Science Center (Ethics Approval No: A2022045). Female Sprague–Dawley rats were randomly divided into three groups (Control, PCOS, and PCOS + electroacupuncture (EA), n = 6/group) and housed under controlled conditions (21–22 ºC, 55–65% humidity, 12–12 h light–dark cycle) with free access to normal chow and water. The study employed 4 week-old rats obtained from Beijing Vital River Laboratory Animal Technology Co., Ltd.

### Establishment of the PCOS-like rat model

Slow-release silicon tube with dihydrotestosterone (DHT, 7.5 mg, daily release amount of 83ug, inner diameter and outer diameter of 0.078″ (ID) and 0.125″ (O.D), 3 cm length) were implanted subcutaneously in the dorsal neck of 21 days of age rats anesthetized by intraperitoneal injection, 0.5 cm from the paraspinal column. Suture after ensuring the location of the embedded silicon tube did not affect the normal feeding and activities of the rats. Closely observed the wound healing and weighed weekly.

### Vaginal smears

The stage of the estrous cycle was determined by microscopic analysis of the predominant cell type in vaginal smears. Seven consecutive days after one month of modeling, the last four consecutive days of the acupuncture intervention, and seven consecutive days prior to sampling, vaginal wall cells were taken on a saline moistened cotton swab and applied flat on slides. Cells were fixed and then stained using Shorr's stain and observed under a microscope to determine the estrous cycle. The proestrus stage was typified by predominantly nucleated cells, the estrus stage exhibited cornified squamous epithelial cells, the metestrus stage was marked by a combination of cornified cells and leukocytes, and the diestrus stage showed a predominance of leukocytes.

### Oral glucose tolerance test (OGTT) and insulin tolerance test (ITT)

Six weeks after the EA intervention, the rats were fasted for 12 h and fasting blood glucose was measured (0 min) using an ACCU-CHEK Performa (Roche), 2 g/kg of 50% Glucose was administered by gastric gavage, and blood glucose was measured at each time point for 15, 30, 45, 60, 90 and 120 min thereafter. And 7 weeks after the EA intervention, the rats were fasted for 8 h and fasting blood glucose was measured (0 min). Then, insulin at a dose of 0.75 IU/kg was injected, and blood glucose was measured at 15, 30, 45, 60, 90 and 120 min.

### EA intervention

Our study employed a consistent set of acupoints in both clinical trials and animal experiments, comprising Zhongwan (CV12), Tianshu (ST25), Guanyuan (CV4), Guilai (ST29), Zhongji (CV3), Zusanli (ST36), and Sanyinjiao (SP6). The electroacupuncture protocol was developed based on previous research by Elisabet Stener-Victorin et al. [31, 32], ensuring methodological continuity between clinical and mechanistic settings. Zhongwan (CV12) was located on the anterior midline, 4 cun above the umbilicus, Tianshu (ST25) was located on 2 cun lateral to the centre of the umbilicus, Guanyuan (CV4) was located on the anterior midline, 3 cun below the umbilicus; Guilai (ST29) was located 4 cun below the umbilicus, 2 cun lateral to Zhongji (CV3), Zhongji (CV3) was located on the anterior midline, 4 cun below the umbilicus, Zusanli (ST36) was located on the lateral side of the knee joint, approximately 2 mm below the fibular head, Sanyinjiao (SP6) was located 3 cun directly above the tip of the medial malleolus, posterior to the medial border of the tibia. After established a PCOS model (1 month), rats in the PCOS + EA group were fixed and treated with low-frequency EA at 9 weeks of age. The treatment is scheduled from 8 a.m. to 10 a.m., once every other day, three times a week, and it lasted for 8 weeks. On CV12, ST25, ST29 and CV3, the needles were attached to an electrical stimulator (HANS-LH202, Huayang Co., Ltd, China) for 20 min at 2 Hz, 2 mA and 10 min at 2 Hz, 3 mA. These needles, measuring 0.20 mm in diameter and 10 mm in length, were sourced from Wujiang Yunlong Medical Instruments Co., Ltd., China. Rats in both control group and PCOS model group were placed inside holder using similar methods but did not receive any treatment during the EA period. Throughout the entire treatment process, all rats remained alert without exhibiting any signs of pain or distress.

### Measurement of hormones

Upon completion of the 12-week experimental protocol, all rats were fasted for eight hours and then euthanized. Terminal blood collection was performed through retro-orbital venous plexus puncture using heparinized capillary tubes, with whole blood immediately transferred to EDTA-coated vacutainers (BD Biosciences, Franklin Lakes, NJ). Subsequent serum isolation was achieved through dual-phase centrifugation to ensure platelet-poor supernatant, with aliquots cryopreserved at − 80 °C until batch analysis.

Fasting insulin levels was measured using radioimmunoassay kit (Beijing North Institute of Biological Technology, Beijing, China). The levels of testosterone, SHBG were determined with 125-I labeled radioimmunoassay kits (Beijing North Institute of Biological Technology, Beijing, China). All kits contained standard samples for quality control and were used according to the manufacturer’s instructions.

### RNA isolation and quantitative real-time PCR

Total RNA from liver tissue was extracted using the MolPure^®^ Cell/Tissue Total RNA kit and reverse transcribed to cDNA using the PrimeScript RT kit. qPCR was performed using the ChamQ Universal SYBR qPCR Master Mix (Vazyme, order Q711-02) on a Line-Gene 9600 instrument. qPCR primers were as follows: [Sequence (5′- > 3′)], [Sequence (5′- > 3′)]. qPCR primers were as follows: [Sequence (5′- > 3′)].

FXR:

Forward primer: CTCCTCTCGTCCTATTATTCC

Reverse primer: TCAGCCAACATTCCCATC

β-Actin:

Forward primer: GCCACCAGTTCGCCATGGAT

Reverse primer: CCCACGATGGGAGGGGAAGA

PCR amplification conditions were as follows: initial denaturation at 95 ℃ for 30 s, 40 cycles of amplification, denaturation at 95 ℃ for 15 s, annealing at 60 ℃ for 30 s, and extension at 60 ℃ for 30 s. Melt curve analysis was performed after the reaction to determine the homogeneity of amplicons.

### RNA-seq analysis

Total RNA was extracted from the livers of rats in the control, the PCOS and the PCOS-EA group. RNA-seq library preparation was performed by Biomarker Technologies. Sequencing libraries were constructed and paired-end mRNA-seq data with read lengths of 150 bp were generated, with two or three biological replicates for each cell line. The raw reads were first aligned to the rat reference genome (mm10) using the STAR software [22] with default parameters. The expression matrix was generated by FeatureCounts software [22]. Gene expression (DEGs) were calculated using the R package DEseq [23]. Expression levels were represented as Fragments Per Kilobase of exon model per Million mapped fragments (FPKM). DEGs were identified based on the following criteria: absolute fold change > 1.5 and the False Discovery Rate (FDR) < 0.05. To compare the functions of the DEGs across the five cell types, we used the Metascape website(https://metascape.org/gp/index.html#/main/step1). In addition, we retrieved all FXR-related genes from the GeneCards search results and determined the intersection between the differentially expressed genes identified in the three groups and those associated with the FXR pathway.

### Statistical analysis

The statistical analyses will be performed by qualified statisticians and biostatisticians. The data in the RCT will be analysed according to the intent-to-treat principle to investigate the differences between the groups.

Clinical outcome measures: continuous variables will be presented as means ± SD and categorical variables as medians with IQRs. Between-group comparisons will be carried out with changes from baseline to after treatment and from baseline to follow-up by ANOVA. P values comparing different groups have been computed using the Mann–Whitney U test with Benjamini-Hochberg (BH) correction.

All statistical analyses of the data will be performed using the SPSS program V.23.0 or higher, and a p value < 0.05 will be considered statistically significant in the RCT and animal experiments. All tests are two-sided and adjustments for multiple comparisons will be performed.

### Data management and quality control of data

We use both paper CRF and web-based eCRF to manage individual participant data. Quality control is handled at two levels. First, the investigators are required to ensure the accuracy when imputing data into the eCRF. Second, data monitoring and validation will be carried out by an independent person not involved in data collection.

## Results

### Serum bile acid profiling of patients with PCOS under acupuncture treatment

**Fig. 1:**
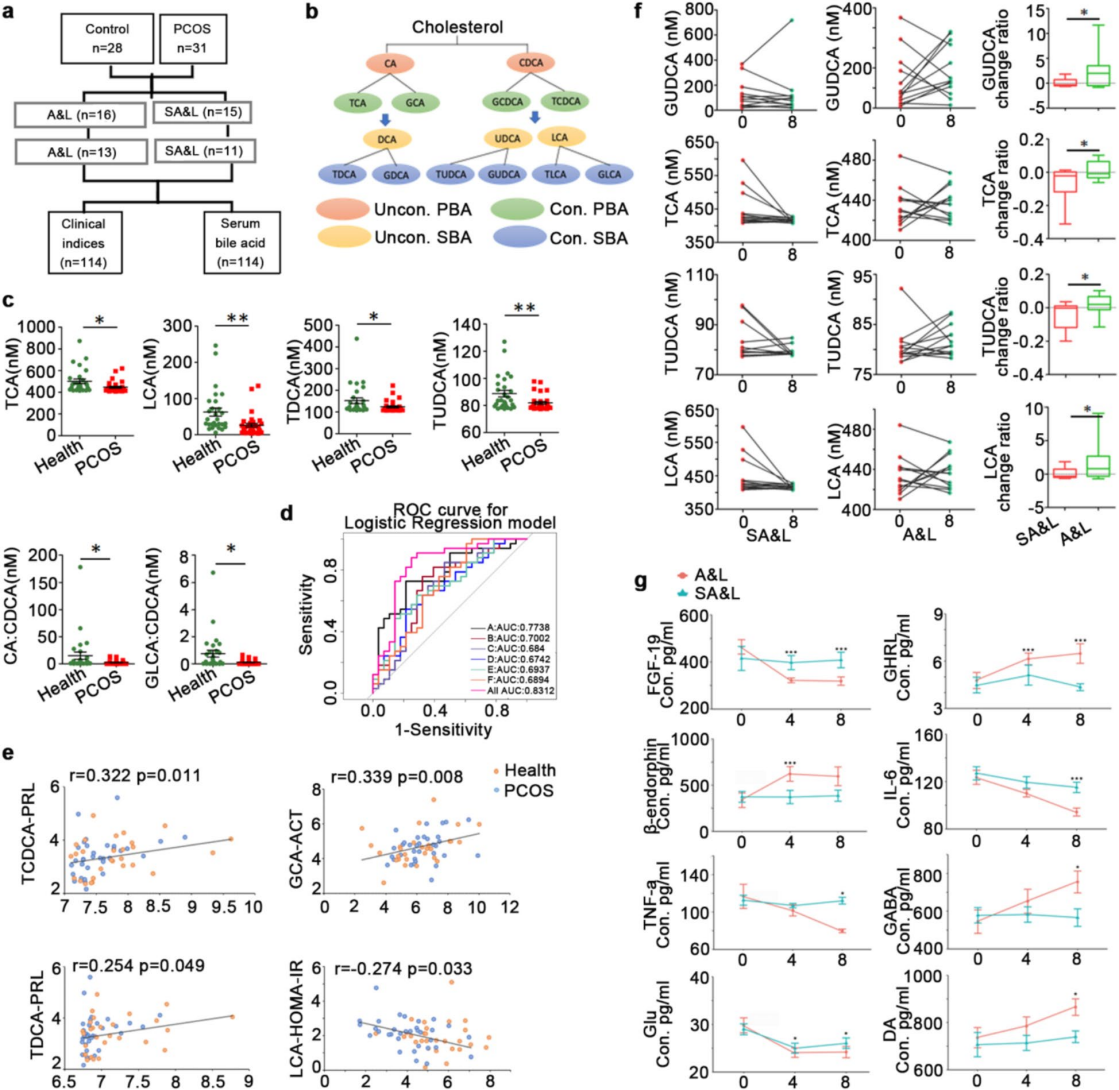
Serum bile acid profiling in Participants. **a** Study cohort overview. The study included 4 months of treatment with acupuncture or with sham acupuncture, accompanied by lifestyle management, and 4 months of follow-up with no intervene. 33 PCOS participants were randomly assigned into the active acupuncture (A&L) or the sham acupuncture (SA&L). All participants received lifestyle management. Serum samples for bile acid analysis were taken before treatment, after 4 months treatment and after 4 month follow up. **b** Diagram depicting the bile acid synthesis and cholesterol clearance pathway. **c** Levels of indicated serum bile acids and ratio between bile acids in PCOS and healthy controls. P values were determined by two-tailed t-test and data are presented as medians with SEM (*p < 0.05; **p < 0.01). **d** Receiver operating characteristic (ROC) curves of differentially expressed bile acids, highlighting a panel of 6 bile acid features using the criteria of adjust *p* < 0.05 between participants with PCOS and the controls. **e** Scatterplot illustrating correlations between selected bile acid concentrations and clinical indicators with Pearson correlation coefficients. **f** Display of specific serum bile acid concentrations in paired untreated PCOS participants versus those who underwent 4 months of acupuncture treatment and a 4-month follow-up. The change rate, calculated by the formula |Treated–untreated|/untreated, is depicted as a box plot (*p < 0.05). **g** Serum concentrations of FGF-19, GHRL, beta-endorphin, IL-6, TNF-α, GABA, Glu and DA across three time points (pre-treatment, post 4 month treatment, and post 4 month follow-up) determined by ELISA (*p < 0.05; ***p < 0.001)

In this study, a total of 408 women were screened, of whom 368 were excluded based on the eligibility criteria. Consequently, 31 patients diagnosed with PCOS and 28 BMI-matched healthy controls were enrolled. The 31 PCOS patients were randomly assigned to receive either A&L or SA&L over a 4 month period. Ultimately, 24 patients (11 in the A&L group and 13 in the SA&L group) completed the follow-up (Fig. 1a).

At the end of 4 months of intervention, the A&L group showed significant symptom improvement, the clinical indices of healthy controls and PCOS women before and after the 4-month intervention are presented in Table 1. In the A&L group, BMI and waist-to-hip ratio were decreased significantly after the intervention compared with baseline (BMI: PCOS 30.18 ± 3.48 vs A&L 27.57 ± 2.95; p < 0.05), whereas no significant change was observed in the SA&L group. Key indices of insulin resistance, including glycated hemoglobin (HbA1c) (PCOS 5.63 ± 0.38 vs A&L 4 months 5.23 ± 0.28, p < 0.01), the Homeostatic Model Assessment for Insulin Resistance HOMA-IR (PCOS 4.48 ± 2.11 vs A&L 4 months 2.89 ± 1.27, p < 0.05), Fasting insulin (PCOS 5.11 ± 0.70 vs A&L 4 months 12.59 ± 4.94, p < 0.05) and the AUC for glucose tolerance (PCOS 1032.40 ± 171.30 vs A&L 4 months 841.39 ± 169.76, p < 0.01), AUC for insulin tolerance (PCOS 15214.64 ± 6943.73 vs A&L 4 months 9766.69 ± 4094.65, p < 0.05) showed notable improvement, paralleled by favorable changes in hyperandrogenism-related markers (SHBG (PCOS 21.86 ± 12.91 vs A&L 4 months 32.36 ± 17.22, p < 0.01), AMH (PCOS 7.62 ± 4.40 vs A&L 4 months 4.92 ± 3.88, p < 0.05) and the FAI (PCOS 12.76 ± 6.46 vs A&L 4 months 7.46 ± 4.32, p < 0.05)). Notably, the therapeutic benefits of A&L treatment persisted beyond the intervention period. At the 4 month follow-up, PCOS in the A&L group maintained significantly reduced BMI, HbA1c, with measurements closely matching those at the end of the intervention. Furthermore, improvements in SHBG, fasting insulin, AUC for glucose tolerance and HOMA-IR remained evident compared with baseline, highlighting the durability of the metabolic and endocrine benefits associated with A&L therapy, suggesting these beneficial effects persisted throughout the 4 month post-treatment follow-up (Table 1).

**Table 1:** Characteristics and clinical indices of 59 participants

Characteristics	Non-PCOS	PCOS	A&L	A&L	SA&L	SA&L	Non-PCOS VS PCOS	A&L 4 months VS PCOS	A&L 8 months VS PCOS	SA&L 4 months VS PCOS	SA&L 8 months VS PCOS
			4 months	8 months	4 months	8 months					
Age (years)	31.07 ± 5.36	28.45 ± 5.06	28.69 ± 5.24	29.23 ± 5.21	28.20 ± 5.03	29.45 ± 4.76	NS	NS	NS	NS	NS
BMI (kg/m^2^)	28.65 ± 2.53	30.18 ± 3.48	27.57 ± 2.95	27.01 ± 2.86	29.80 ± 3.84	28.79 ± 3.94	NS	*	*	NS	NS
Waist-hip ratio (%)	87.68 ± 5.49	87.30 ± 5.67	85.55 ± 5.98	85.11 ± 5.84	87.14 ± 6.08	87.00 ± 6.56	NS	NS	NS	NS	NS
Body fat rate (%)	40.11 ± 3.69	42.17 ± 5.23	38.81 ± 4.94	36.30 ± 6.75	40.02 ± 6.28	39.24 ± 6.76	NS	NS	*	NS	NS
Lean weight	44.21 ± 3.66	44.30 ± 2.95	43.16 ± 2.51	43.87 ± 3.99	44.09 ± 3.28	44.51 ± 3.24	NS	NS	NS	NS	NS
HbA1c	5.50 ± 0.55	5.63 ± 0.38	5.23 ± 0.28	5.13 ± 0.40	5.48 ± 0.31	5.58 ± 0.35	NS	**	**	NS	NS
Fasting Glucose (mmol/L)	5.38 ± 0.76	5.11 ± 0.70	5.09 ± 0.40	5.30 ± 0.92	5.29 ± 0.56	5.35 ± 0.77	NS	NS	NS	NS	NS
Fasting insulin (mU/L)	19.06 ± 15.22	19.45 ± 8.14	12.59 ± 4.94	14.30 ± 6.95	15.81 ± 6.7	13.98 ± 8.72	NS	*	*	NS	*
HOMA-IR	4.95 ± 5.98	4.48 ± 2.11	2.89 ± 1.27	3.44 ± 1.84	3.72 ± 1.58	3.28 ± 2.08	NS	*	*	NS	NS
HOMA-B	202.35 ± 108.34	394.76 ± 721.38	159.97 ± 50.87	174.44 ± 79.84	190.19 ± 92.59	172.92 ± 103.24	NS	NS	NS	NS	NS
AUC 120 glucose	990.75 ± 263.23	1032.40 ± 171.30	841.39 ± 169.76	878.10 ± 128.58	947.50 ± 216.41	847.67 ± 103.10	NS	**	*	NS	**
AUC 120 insulin	12081.32 ± 7666.52	15,214.64 ± 6943.73	9766.69 ± 4094.65	9792.95 ± 4037.83	11,159.30 ± 6049.71	12,111.54 ± 7357.05	NS	*	*	*	NS
PRL ng/ml	11.73 ± 5.93	12.32 ± 8.39	12.70 ± 8.14	11.62 ± 6.08	14.37 ± 10.19	13.51 ± 11.74	NS	NS	NS	NS	NS
LH mIU/ml	4.50 ± 2.99	7.40 ± 4.66	5.82 ± 3.24	5.46 ± 3.10	5.78 ± 2.67	8.69 ± 4.42	*	NS	NS	NS	NS
FSH mIU/ml	6.79 ± 2.81	5.51 ± 1.49	6.31 ± 1.27	5.92 ± 1.01	5.44 ± 1.24	6.07 ± 1.99	NS	NS	NS	NS	NS
E2 pmol/L	178.20 ± 50.77	200.19 ± 106.80	152.69 ± 38.86	142.00 ± 55.71	166.01 ± 56.38	161.54 ± 43.82	NS	NS	NS	NS	NS
PRG nmol/L	1.74 ± 2.95	1.04 ± 0.53	0.98 ± 0.40	0.92 ± 0.34	0.99 ± 0.36	0.90 ± 0.30	NS	NS	NS	NS	NS
Androstendione nmol/L	7.27 ± 3.28	10.13 ± 4.58	8.96 ± 4.84	8.36 ± 4.52	10.43 ± 4.50	10.46 ± 3.27	*	NS	NS	NS	NS
Testosterone nmol/L	1.55 ± 0.50	2.29 ± 0.82	1.99 ± 0.91	2.17 ± 0.95	2.19 ± 0.74	2.13 ± 0.71	*	NS	NS	NS	NS
FAI	6.29 ± 4.03	12.76 ± 6.46	7.46 ± 4.32	8.78 ± 5.21	12.60 ± 6.75	9.97 ± 4.23	***	*	NS	NS	NS
SHBG nmol/L	31.70 ± 14.64	21.86 ± 12.91	32.36 ± 17.22	29.16 ± 10.35	21.51 ± 10.97	24.13 ± 7.70	**	**	**	NS	NS
AMH ng/ml	3.83 ± 3.59	7.62 ± 4.40	4.92 ± 3.88	6.12 ± 4.48	7.62 ± 5.78	10.37 ± 5.65	**	*	NS	NS	NS

P values comparing different groups have been computed using the Mann–Whitney U test with Benjamini-Hochberg (BH) correction. A&L: Acupuncture and lifestyle treatment, SA&L: lifestyle treatment only *P < 0.05, **P < 0.01, ***P < 0.001

To explore the potential mechanisms underlying the effects of acupuncture, clinical data were collected from 31 patients with PCOS before the intervention, from the same patients after 4 months of acupuncture treatment, from 28 healthy controls, and from 24 patients with PCOS who completed the 4 month follow-up. Plasma samples from these participants were subjected to bile acid analysis. Serum bile acid profiling revealed pronounced reductions in TCA (Healthy: 501.59 nM ± 109.40 nM vs PCOS: 446.33 nM ± 49.37 nM), LCA (Healthy: 62.59 nM ± 26.11 vs PCOS: 26.11 nM ± 30.20 nM), TDCA (Healthy: 151.98 nM ± 69.82 nM vs PCOS: 123.73 nM ± 25.46 nM), and TUDCA (Healthy: 88.65 nM ± 12.80 nM vs PCOS: 81.79 nM ± 5.53 nM) levels in PCOS participants (Fig. 1b, c). A decreased CA to CDCA ratio (Healthy: 15.12 ± 35.46 vs PCOS: 2.43 ± 3.71) suggested a shift from primary to secondary bile acid synthesis pathways, while altered GLCA to CDCA ratio (Healthy: 0.75 ± 1.38 vs PCOS: 0.11 ± 0.17) indicated dysregulated secondary bile acid metabolism. Logistic regression confirmed these bile acid profiles as discriminators between PCOS women and healthy controls (Fig. 1d). Notably, LCA—a microbiota-derived bile acid that enhances insulin sensitivity via TGR5-mediated glucagon-like peptide-1 (GLP-1) secretion—showed a negative correlation with HOMA-IR (Fig. 1e). This aligns with established mechanisms linking bile acid signaling to insulin resistance in PCOS [24, 25].

Furthermore, TDCA and TCDCA correlated with PRL, while GCA levels associated with ACTH, implicating bile acids in neuroendocrine regulation. Compared to sham controls, 4 month acupuncture followed by 4 month observation significantly reversed serum levels of GUDCA, TUDCA, TCA, and LCA according to the change rate comparing 8 months to baseline (Fig. 1f). This is consistent with prior evidence: TUDCA improves glucose tolerance and reduces adiposity, while metformin elevates TUDCA in insulin-resistant models [26]. Similarly, GUDCA enhances insulin sensitivity in diabetic cohorts, corroborating acupuncture’s efficacy in metabolic modulation [9].

Downstream molecular targets were quantified via enzyme-linked immunosorbent assay (ELISA), while GABA and DA levels were measured using UPLC-MS/MS. Acupuncture treatment significantly reduced serum fibroblast growth factor 19 (FGF-19) levels, suggesting suppression of intestinal FXR signaling—a pathway implicated in hepatic metabolic regulation and recognized as a therapeutic target for metabolic disorders [9]. This aligns with prior evidence that acupuncture modulates FXR activity (Fig. 1g).

Notably, the acupuncture-treated group exhibited marked alterations in neuroendocrine and immune markers compared to sham controls (Fig. 1g). β-endorphin, DA, and GABA levels increased significantly, whereas Glu decreased. Given that β-endorphin, DA, and GABA inhibit gonadotropin-releasing hormone (GnRH) secretion, while Glu stimulates it, these shifts suggest that acupuncture may regulate GnRH-mediated ovulatory dysfunction in PCOS [5]. Concurrent reductions in interleukin-6 (IL-6) and tumor necrosis factor-α (TNF-α) further indicate acupuncture’s anti-inflammatory effects.

Additionally, elevated ghrelin (GHRL) levels post-acupuncture correlated with improved insulin sensitivity, consistent with studies linking GHRL to insulin resistance mitigation in PCOS [7]. These findings collectively highlight acupuncture’s multi-target regulatory effects on metabolic, neuroendocrine, and inflammatory pathways in PCOS.

### Electroacupuncture ameliorates PCOS rat model and modulate hepatic FXR signaling

**Fig. 2:**
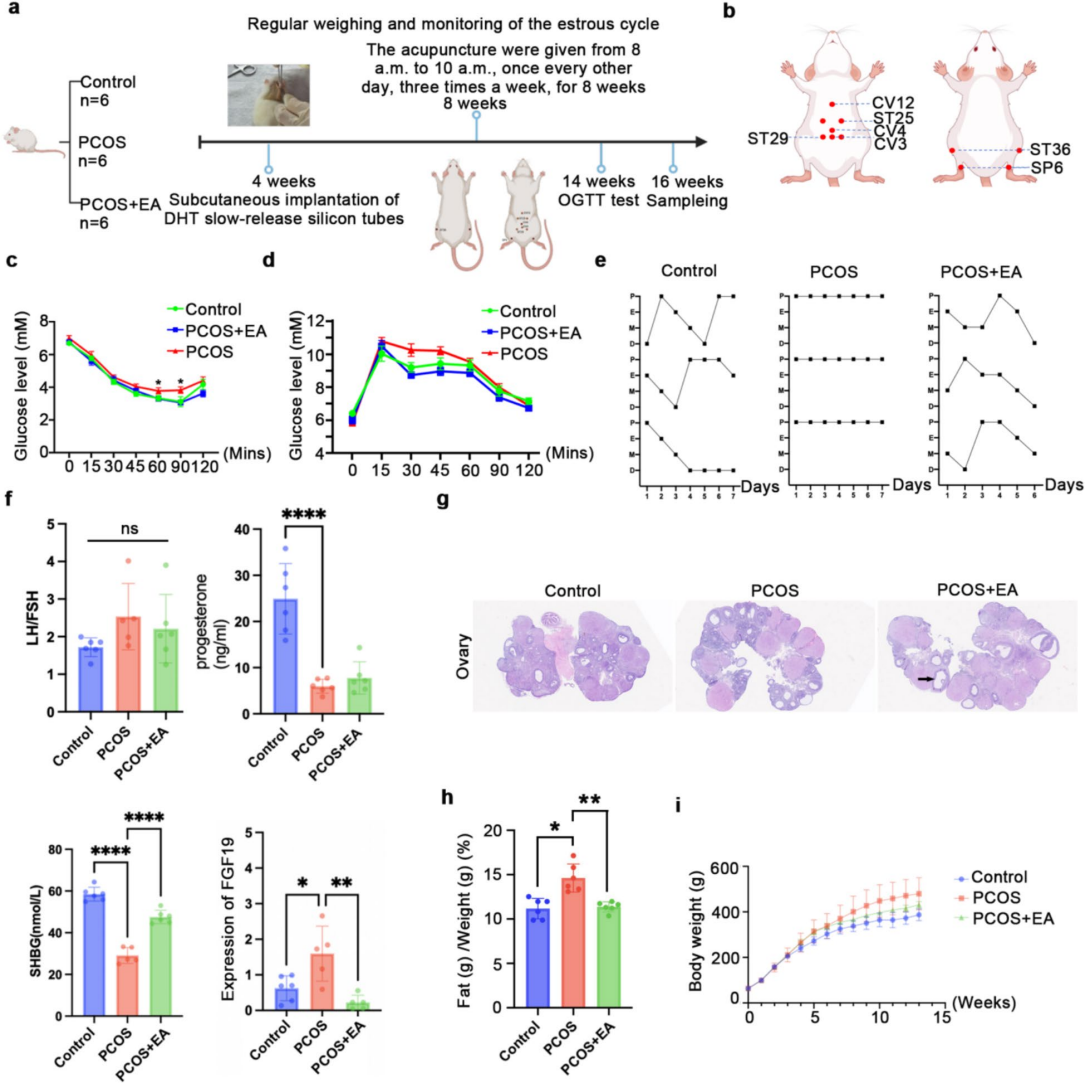
Phenotype of DHT-induced PCOS rats after electroacupuncture treatment. **a** Schematic representation of electroacupuncture treatment and the experimental procedure in DHT-induced PCOS rats. **b** Schematic diagram of electroacupuncture at acupoints in rats. **c** Blood glucose levels of OGTT (*p < 0.05; **p < 0.01). **d** Blood glucose levels of itt (*p < 0.05; **p < 0.01). **e** The estrous cycle of each rat was detected for 7 consecutive days. **f** The concentrations of serum hormones, including FSH, LH, progesterone, and SHBG, were measured using ELISA (****p < 0.0001). n = 6. The expression level of FGF19 was detected by qRT-PCR (*p < 0.05; **p < 0.01). n = 3. **g** HE staining of ovarian sections. **h** The percentage of fat against total rates of rat models (n = 6). **i** The weighing of the body weight of rats. n = 6

To validate the clinical findings, we established a rat model of PCOS through the subcutaneous implantation of a slow-release silicone tube containing dihydrotestosterone (DHT) [27, 28]. This model was subsequently treated with manual acupuncture combined with electroacupuncture [18, 29]. Glucose tolerance and insulin sensitivity in a PCOS rat model was assessed using OGTT and ITT. Compared with the control group, the PCOS group exhibited a significantly higher glucose, indicating impaired glucose metabolism. The acupuncture treatment group showed no significant difference in glucose compared to the control, suggesting improved glucose regulation (Fig. 2c and d). To investigate the mechanism underlying acupuncture-mediated improvement of insulin resistance, we employed qPCR to measure the expression levels of fibroblast growth factor 19 (FGF19), a gene associated with the FXR signaling pathway. PCOS rats displayed significant FGF19 upregulation compared to controls, which was reversed after acupuncture (Fig. 2f). Reproductive evaluation identified irregular estrous cycles (Fig. 2e) and ovarian histopathological changes characterized by an abundance of immature and atretic follicles in PCOS rats, indicative of sporadic ovulation (Fig. 2g). Hormonal profiling further revealed elevated LH/FSH ratios accompanied by markedly reduced progesterone and SHBG levels in the PCOS group (Fig. 2f). Acupuncture significantly alleviated the phenotypic manifestations of the PCOS model. Notably, the reduction in SHBG, indicative of sex hormone receptor downregulation or insensitivity—a diagnostic hallmark of PCOS—was reversed following electroacupuncture treatment. The model group exhibited an increase in fat content (Fig. 2h) and body weight (Fig. 2i) compared to the control group, which was attenuated following acupuncture treatment. These findings suggest that acupuncture therapy effectively alleviates obesity, reproductive endocrine disturbances, and insulin resistance in PCOS through modulation of the FXR signaling pathway.

### Liver transcriptome analysis in a PCOS rat model treated with electroacupuncture revealed the modulation of bile acid metabolism

**Fig. 3:**
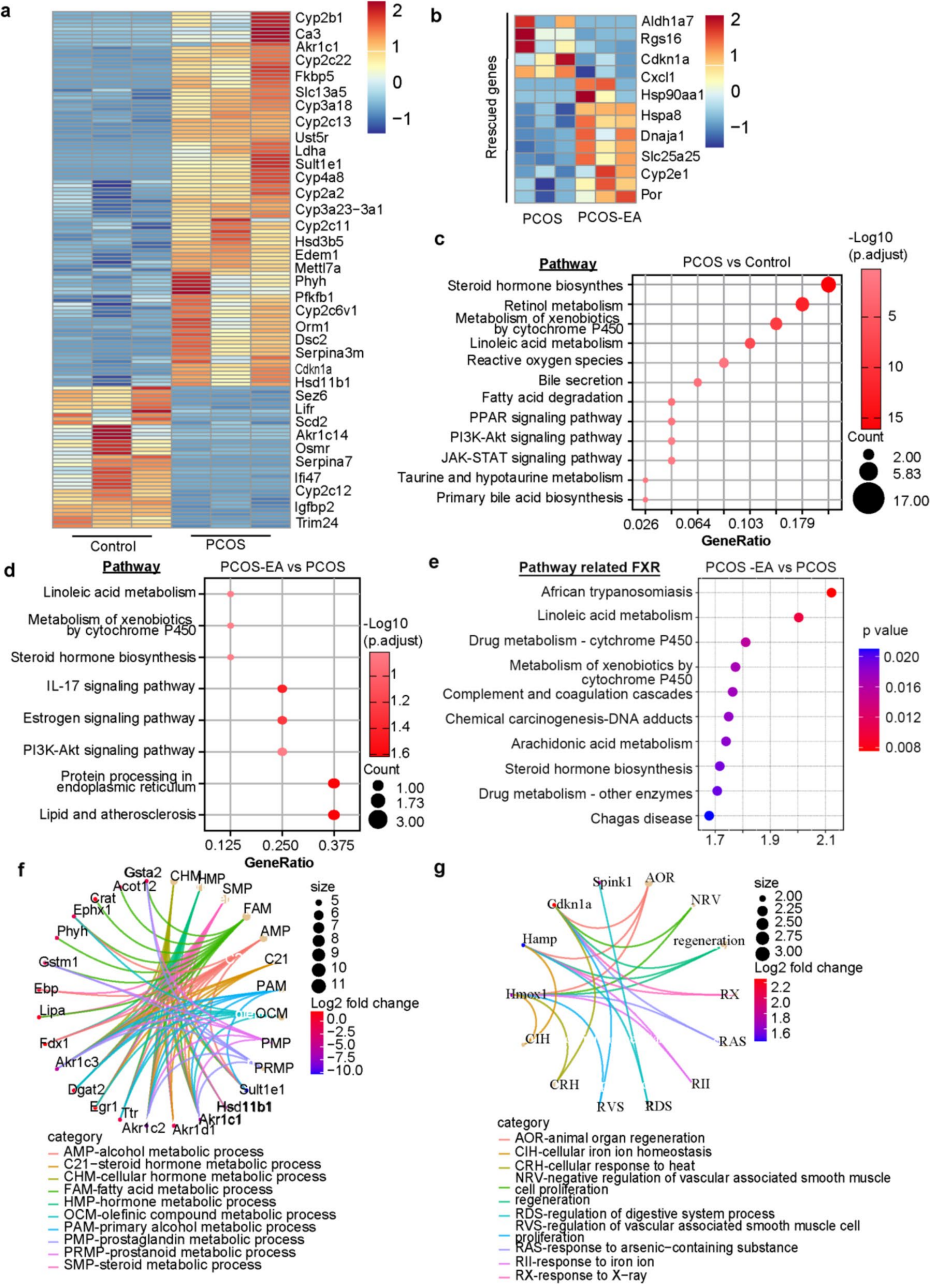
Liver bulk RNA-Seq Analysis of DHT-Induced PCOS Rat and acupuncture treated PCOS rat. **a** Differential gene expression analysis between Control and PCOS. Red indicated upregulated genes (fold change > 1.5, FDR < 0.05); Blue indicates downregulated genes (fold change > 1.5 and FDR < 0.05). **b** Differential gene expression analysis between PCOS-EA and PCOS. Red indicated upregulated genes (fold change > 1.5, FDR < 0.05); Blue indicates downregulated genes (fold change > 1.5 and FDR < 0.05). **c** Enrichment of signaling pathways related to upregulated and downregulated genes in Control and PCOS, as analyzed through Metascape. **d** Enrichment of Signaling pathways related to upregulated and downregulated genes in PCOS-EA and PCOS, as analyzed through Metascape. **e** Enrichment analysis of FXR-related signaling pathways among DEGs in PCOS-EA and PCOS. **f** Enrichment of biological process related to bile-acid of DEGs in Control and PCOS, as analyzed through Metascape. **g** Enrichment of biological process related to bile-acid of DEGs in PCOS-EA and PCOS, as analyzed through Metascape

Liver sample bulk RNA-seq was performed to compare hepatic transcriptomes between control rats, untreated PCOS rats and PCOS rats treated with electroacupuncture (PCOS-EA). Differential DEGs analysis revealed significant upregulation of multiple genes including *Cyp2b1*, *Fkbp5*, *Cdkn1a*, *Osmr*, *Igmbp2* in PCOS versus controls (Fig. 3a). KEGG enrichment analysis of DEGs highlighted dysregulation in pathways critical to hormone synthesis and bile acid metabolism, including steroid hormone biosynthesis, cytochrome P450-mediated xenobiotic metabolism, bile secretion, primary bile acid biosynthesis, and PI3K-Akt signaling (Fig. 3c). These findings align with prior evidence linking bile acid biosynthesis inhibition to PCOS pathogenesis [9], suggesting impaired fatty acid metabolism and bile acid homeostasis in PCOS.

Electroacupuncture reversed the expression of multiple DEGs in PCOS rats (Fig. 3b). KEGG pathway analysis demonstrated restored activity in linoleic acid metabolism, xenobiotic metabolism, steroid hormone biosynthesis, and PI3K-Akt signaling (Fig. 3d). Pathways involved in linoleic acid metabolism, steroid hormone biosynthesis, and xenobiotic metabolism are closely linked to the FXR signaling pathway (Fig. 3e). Gene Ontology (GO) enrichment (f—PCOS vs Control, g—PCOS EA vs PCOS) identified acupuncture-induced modulation of bile acid-associated biological processes (Fig. 3f–g). Collectively, these results indicate that electroacupuncture ameliorates bile acid dysmetabolism in PCOS.

## Discussion

This study demonstrated that acupuncture treatment significantly improved insulin resistance, endocrine profiles, and body mass index (BMI) in PCOS women, with sustained therapeutic effects observed at the 16 week follow-up (no rebound detected). Moreover, acupuncture modulated serum bile acid profiles, concomitant with decreased circulating levels of FGF19. Consistent with human findings, in a PCOS rat model, acupuncture significantly ameliorated insulin resistance, hyperandrogenism, and adiposity. Meanwhile, acupuncture altered hepatic expression of genes associated with bile acid metabolism and the FXR signaling pathway in PCOS rats. Collectively, these findings suggest that acupuncture alleviates insulin resistance in PCOS by restoring bile acid homeostasis, likely mediated through the bile acid-FXR axis.

Acupuncture has been proposed as an insulin sensitizer with potential benefits for managing obesity and type 2 diabetes [30, 31]. In the present study, acupuncture combine significantly reduced HOMA-IR, consistent with findings from previous non-randomized studies [30, 32]. Notably, previous work by Stener-Victorin et al. (n = 21) demonstrated that a single acupuncture session could enhance whole-body glucose uptake through activation of the sympathetic and partial parasympathetic nervous systems [33]. Furthermore, acupuncture improved glucose metabolism, as evidenced by regulating the gut microbiota, bile acids, whose metabolism is mediated by gut microbiota, play a critical role in the pathogenesis of PCOS [9]. In patients with PCOS, specific bile acids—including TCA, LCA, TDCA, and TUDCA—were significantly reduced, alongside decreased ratios of CA/CDCA and GLCA/CDCA. These alterations revealed a distinct bile acids profile in PCOS compared with healthy individuals, supporting the potential utility of the bile acids spectrum as a physiological biomarker for distinguishing the two groups. Previous studies [24, 25] have shown that LCA can enhance insulin sensitivity via activation of the TGR5-mediated GLP-1 pathway. In agreement with these reports, our study identified a negative correlation between circulating LCA levels and HOMA-IR, suggesting an association between bile acids pathway and insulin resistance in PCOS.

Further comparative analysis of serum bile acids levels between SA&L and A&L group, acupuncture significantly restored GUDCA, TUDCA, TCA, and LCA levels. Notably, these beneficial effects persisted for up to four months post-treatment. Metformin has been reported to improve glucose tolerance, ameliorate insulin resistance, and reduce obesity by increasing TUDCA levels, while GUDCA has also been shown to improve insulin sensitivity in diabetic populations [9, 34]. In our study, serum FGF19 levels were significantly reduced following acupuncture, implicating modulation of the FXR signaling pathway in alleviating insulin resistance in PCOS patients [35]. Bile acid synthesis is under negative feedback control through activation of the nuclear receptor FXR in the ileum and liver [36]. Collectively, these results suggest that acupuncture improves insulin sensitivity in PCOS through regulation of the bile acids profile and reducing the level of FGF19.

In addition to metabolic effects, marked changes in neuroendocrine and immune markers were observed in the A&L group compared with SA&L group. β-Endorphin, DA, and GABA are known to inhibit GnRH secretion, whereas Glu stimulates it. This pattern suggests that acupuncture may correct GnRH-mediated endocrine dysregulation in PCOS by modulating the secretion of β-endorphin, DA, GABA, and Glu [5]. Concurrent decreases in interleukin-6 (IL-6) and TNF-α further support the anti-inflammatory properties of acupuncture. Moreover, increased ghrelin (GHRL) levels were associated with improved insulin sensitivity, consistent with previous findings that ghrelin attenuates insulin resistance in women with PCOS [7]. Taken together, these findings suggest that acupuncture exerts multi-target regulatory effects on metabolic, neuroendocrine, and inflammatory pathways in women with PCOS.

Consistent with clinical findings, experiments in a DHT-induced PCOS rat model demonstrated that acupuncture significantly improved insulin resistance, estrous cyclicity, reduced body fat, increased serum SHBG levels and changed ovarian FGF19 expression compared to PCOS, indicating acupuncture effectively mitigates endocrine disorder and insulin resistance in PCOS pathophysiology. The phenotypic improvements observed here are consistent with those reported in previous studies [37–40].

In PCOS, unsaturated fatty acid metabolism, sterol metabolism, and bile acid metabolism form a complex regulatory network mediated by the generation of secondary bile acids (e.g., deoxycholic acid, DCA). This suppression mechanistically leads to dual activation of both the farnesoid X receptor (FXR) and Takeda G protein-coupled receptor 5 (TGR5) signaling pathways, which collectively regulate the expression of sterol metabolism-associated genes [13, 41]. Concurrently, hyperandrogenemia reduces the bile acids pool by downregulating hepatic bile acids synthesis enzymes, including CYP7A1. This suppression alters gut microbial composition (e.g., decreasing Bifidobacterium abundance), thereby creating a positive feedback loop that exacerbates metabolic dysregulation [36]. Given the liver’s central role in bile acids metabolism, we performed transcriptome profiling of hepatic tissue from rats. Differentially expressed genes in the livers of PCOS rats, compared with controls, were predominantly enriched in pathways such as steroid hormone biosynthesis, linoleic acid metabolism, taurine and hypotaurine metabolism, fatty acid degradation, and the PI3K–AKT signaling pathway—findings in agreement with earlier reports [42–45]. Acupuncture treatment significantly reversed alterations in steroid hormone biosynthesis, linoleic acid metabolism, and PI3K–AKT signaling. Additionally, gene enrichment analysis demonstrated that differentially expressed genes in acupuncture-treated PCOS rat livers were associated with FXR-related pathways. Collectively, these results suggest that acupuncture modulates gene expression across the steroid hormone synthesis–metabolic axis, thereby improving insulin resistance and impairments in follicular development in PCOS rats.

Integrating evidence from clinical and animal studies, our findings demonstrate that acupuncture can effectively modulate insulin resistance and metabolic disturbances in women with PCOS by regulating the bile acid–insulin resistance metabolic axis, offering a non-pharmacological therapeutic option for this condition, enhancing understanding of acupuncture’s multi-level regulatory effects on endocrine and metabolic dysfunction, underscoring its potential as a comprehensive intervention. This study has certain limitations. The stringent inclusion and exclusion criteria resulted in a relatively small sample size, which may limit the generalizability of the findings. Building on these insights, future research should focus on developing an optimized and accessible acupuncture protocol suitable for routine clinical application, while elucidating the mechanistic pathways—particularly the interplay among metabolism and PCOS phenotypes, developing an optimized, clinically feasible acupuncture protocol for widespread application.

## Conclusions

In summary, bile acids appear to be pivotal in the etiology of PCOS. Acupuncture offer therapeutic benefits by modulating bile acid metabolism, especially targeting specific bile acids (GUDCA, TCA, TUDCA, LCA). The potential mechanisms might revolve around the influence of acupuncture on bile acid-FXR axis. The bile acids pathway we have identified in this study could serve as promising targets for acupuncture-based interventions in women with PCOS, with the potential to prevent or slow disease progression and offer novel therapeutic options for affected patients.

## Data Availability

Data supporting the findings of this study are not publicly available due to privacy or ethical restrictions. Anonymized data may become available to third parties after request to the email address zoe@bjmu.edu.cn.

## Abbreviations

GDCA: Glycodeoxycholic acid
TUDCA: Tauroursodeoxycholic acid
CDCA: Chenodeoxycholic acid
CA: Cholic acid
LCA: Lithocholic acid
TCA: Taurocholic acid
THDCA: Taurohyodeoxycholic acid
TCDCA: Taurochenodeoxycholic acid
TDCA: Taurodeoxycholic acid
TLCA: Taurolithocholic acid
GCA: Glycocholic acid
GLCA: Glycolithocholic acid
GUDCA: Glycoursodeoxycholic acid
EA: Electroacupuncture
UPLC-MS/MS: Ultra-performance liquid chromatography-tandem mass spectrometry
FXR: Farnesoid X receptor
A2: Androstenedione
SHBG: Sex hormone-binding globulin
FAI: Free Androgen Index
AUC: Area under the curve
PRL: Prolactin
GABA: Gamma amino butyric acid
HbA1c: Glycated hemoglobin
DA: Dopamine
Glu: Glutamate
PCOS: Polycystic ovary syndrome
DHT: Dihydrotestosterone
AMH: Anti-Müllerian hormone
IR: Insulin resistance
LH: Luteinizing hormone
T: Testosterone
FGF-19: Fibroblast growth factor-19
IL-6: Interleukin-6
ACTH: Adrenocorticotropic-hormone
ELISA: Enzyme-linked immunosorbent assay
TNF-a: Tumor Necrosis Factor alpha
GLP-1: Glucagon-like peptide-1

## References

[1] Azziz R. Polycystic Ovary Syndrome. Obstet Gynecol. 2018;132:321–36. 10.1097/AOG.0000000000002698. 29995717 10.1097/AOG.0000000000002698

[2] Teede HJ, Misso ML, Costello MF, Dokras A, Laven J, Moran L, et al. Recommendations from the international evidence-based guideline for the assessment and management of polycystic ovary syndrome. Hum Reprod. 2018;33:1602–18. 10.1093/humrep/dey256. 30052961 10.1093/humrep/dey256 PMC6112576

[3] Lim CED, Ng RWC, Cheng NCL, Zhang GS, Chen H. Acupuncture for polycystic ovarian syndrome. Cochrane Database Syst Rev. 2019;7:CD007689. 10.1002/14651858.CD007689.pub4. 31264709 10.1002/14651858.CD007689.pub4 PMC6603768

[4] Ye Y, Zhou CC, Hu HQ, Fukuzawa I, Zhang HL. Underlying mechanisms of acupuncture therapy on polycystic ovary syndrome: evidences from animal and clinical studies. Front Endocrinol (Lausanne). 2022;13:1035929. 10.3389/fendo.2022.1035929. 36353235 10.3389/fendo.2022.1035929 PMC9637827

[5] Zhang H, Wang W, Zhao J, Jiao P, Zeng L, Zhang H, et al. Relationship between body composition, insulin resistance, and hormonal profiles in women with polycystic ovary syndrome. Front Endocrinol (Lausanne). 2022;13:1085656. 10.3389/fendo.2022.1085656. 36699018 10.3389/fendo.2022.1085656 PMC9869160

[6] Amiri M, Tehrani FR, Bidhendi-Yarandi R, Behboudi-Gandevani S, Azizi F, Carmina E. Relationships between biochemical markers of hyperandrogenism and metabolic parameters in women with polycystic ovary syndrome: a systematic review and meta-analysis. Horm Metab Res. 2019;51:22–34. 10.1055/a-0806-8281. 30650457 10.1055/a-0806-8281

[7] Yang Q, Vijayakumar A, Kahn BB. Metabolites as regulators of insulin sensitivity and metabolism. Nat Rev Mol Cell Biol. 2018;19:654–72. 10.1038/s41580-018-0044-8. 30104701 10.1038/s41580-018-0044-8 PMC6380503

[8] Yue F, Xing L, Wu S, Wei L, Zhou Z, Shi Y, et al. Constant light exposure alters gut microbiota and short-/medium-chain fatty acids and aggravates PCOS-like traits in HFD-fed rats. Obesity (Silver Spring). 2022;30:694–706. 10.1002/oby.23380. 35128797 10.1002/oby.23380

[9] Yun C, Yan S, Liao B, Ding Y, Qi X, Zhao M, et al. The microbial metabolite agmatine acts as an FXR agonist to promote polycystic ovary syndrome in female mice. Nat Metab. 2024;6:947–62. 10.1038/s42255-024-01041-8. 38769396 10.1038/s42255-024-01041-8

[10] Zhang B, Shen S, Gu T, Hong T, Liu J, Sun J, et al. Increased circulating conjugated primary bile acids are associated with hyperandrogenism in women with polycystic ovary syndrome. J Steroid Biochem Mol Biol. 2019;189:171–5. 10.1016/j.jsbmb.2019.03.005. 30849463 10.1016/j.jsbmb.2019.03.005

[11] Sun L, Xie C, Wang G, Wu Y, Wu Q, Wang X, et al. Gut microbiota and intestinal FXR mediate the clinical benefits of metformin. Nat Med. 2018;24:1919–29. 10.1038/s41591-018-0222-4. 30397356 10.1038/s41591-018-0222-4 PMC6479226

[12] Cherney DZI, Lam TKT. A gut feeling for metformin. Cell Metab. 2018;28:808–10. 10.1016/j.cmet.2018.11.012. 30517894 10.1016/j.cmet.2018.11.012

[13] Qi X, Yun C, Sun L, Xia J, Wu Q, Wang Y, et al. Gut microbiota-bile acid-interleukin-22 axis orchestrates polycystic ovary syndrome. Nat Med. 2019;25:1225–33. 10.1038/s41591-019-0509-0. 31332392 10.1038/s41591-019-0509-0 PMC7376369

[14] Tong TT, Bai LB, Yau LF, Li JY, Huang H, Jiang ZH. Pharmacological effects of bile acids on polycystic ovary syndrome via the regulation of chemerin. Chin Med. 2025;20:45. 10.1186/s13020-025-01078-1. 40181388 10.1186/s13020-025-01078-1 PMC11969753

[15] Wang H, Wang Q, Liang C, Su M, Wang X, Li H, et al. Acupuncture regulating gut microbiota in abdominal obese rats induced by high-fat diet. Evid Based Complement Alternat Med. 2019;2019:4958294. 10.1155/2019/4958294. 31275411 10.1155/2019/4958294 PMC6582896

[16] Chen Y, Fan B, Zeng J, Zou Y, Tao C, Chen C, et al. Single-cell RNA transcriptomics and multi-omics analyses reveal the clinical effects of acupuncture on methadone reduction. Research. 2025;8:0741. 10.34133/research.0741. 40556944 10.34133/research.0741 PMC12187353

[17] Stener-Victorin E, Zhang H, Li R, Friden C, Li D, Wang W, et al. Acupuncture or metformin to improve insulin resistance in women with polycystic ovary syndrome: study protocol of a combined multinational cross sectional case-control study and a randomised controlled trial. BMJ Open. 2019;9:e024733. 10.1136/bmjopen-2018-024733. 30612112 10.1136/bmjopen-2018-024733 PMC6326273

[18] Manneras L, Jonsdottir IH, Holmang A, Lonn M, Stener-Victorin E. Low-frequency electro-acupuncture and physical exercise improve metabolic disturbances and modulate gene expression in adipose tissue in rats with dihydrotestosterone-induced polycystic ovary syndrome. Endocrinology. 2008;149:3559–68. 10.1210/en.2008-0053. 18388196 10.1210/en.2008-0053

[19] Matthews DR, Hosker JP, Rudenski AS, Naylor BA, Treacher DF, Turner RC. Homeostasis model assessment: insulin resistance and beta-cell function from fasting plasma glucose and insulin concentrations in man. Diabetologia. 1985;28:412–9. 10.1007/BF00280883. 3899825 10.1007/BF00280883

[20] Hagmar M, Berglund B, Brismar K, Hirschberg AL. Hyperandrogenism may explain reproductive dysfunction in Olympic athletes. Med Sci Sports Exerc. 2009;41:1241–8. 10.1249/MSS.0b013e318195a21a. 19461542 10.1249/MSS.0b013e318195a21a

[21] Iwata M, Maeda S, Kamura Y, Takano A, Kato H, Murakami S, et al. Genetic risk score constructed using 14 susceptibility alleles for type 2 diabetes is associated with the early onset of diabetes and may predict the future requirement of insulin injections among Japanese individuals. Diabetes Care. 2012;35:1763–70. 10.2337/dc11-2006. 22688542 10.2337/dc11-2006 PMC3402252

[22] Liao Y, Smyth GK, Shi W. FeatureCounts: an efficient general purpose program for assigning sequence reads to genomic features. Bioinformatics. 2014;30:923–30. 10.1093/bioinformatics/btt656. 24227677 10.1093/bioinformatics/btt656

[23] Dobin A, Gingeras TR. Mapping RNA-seq reads with STAR. Curr Protoc Bioinformatics. 2015. 10.1002/0471250953.bi1114s51. 26334920 10.1002/0471250953.bi1114s51 PMC4631051

[24] Higgins V, Asgari S, Hamilton JK, Wolska A, Remaley AT, Hartmann B, et al. Postprandial dyslipidemia, hyperinsulinemia, and impaired gut peptides/bile acids in adolescents with obesity. J Clin Endocrinol Metab. 2020;105:1228–41. 10.1210/clinem/dgz261. 31825485 10.1210/clinem/dgz261 PMC7065844

[25] An J, Wang L, Song S, Tian L, Liu Q, Mei M, et al. Electroacupuncture reduces blood glucose by regulating intestinal flora in type 2 diabetic mice. J Diabetes. 2022;14:695–710. 10.1111/1753-0407.13323. 36195536 10.1111/1753-0407.13323 PMC9574722

[26] Zhang Y, Cheng Y, Liu J, Zuo J, Yan L, Thring RW, et al. Tauroursodeoxycholic acid functions as a critical effector mediating insulin sensitization of metformin in obese mice. Redox Biol. 2022;57:102481. 10.1016/j.redox.2022.102481. 36148770 10.1016/j.redox.2022.102481 PMC9493383

[27] Jin J, Ma Y, Tong X, Yang W, Dai Y, Pan Y, et al. Metformin inhibits testosterone-induced endoplasmic reticulum stress in ovarian granulosa cells via inactivation of p38 MAPK. Hum Reprod. 2020;35:1145–58. 10.1093/humrep/deaa077. 32372097 10.1093/humrep/deaa077 PMC7259369

[28] Cui P, Hu W, Ma T, Hu M, Tong X, Zhang F, et al. Long-term androgen excess induces insulin resistance and non-alcoholic fatty liver disease in PCOS-like rats. J Steroid Biochem Mol Biol. 2021;208:105829. 10.1016/j.jsbmb.2021.105829. 33513383 10.1016/j.jsbmb.2021.105829

[29] Wen Q, Hu M, Lai M, Li J, Hu Z, Quan K, et al. Effect of acupuncture and metformin on insulin sensitivity in women with polycystic ovary syndrome and insulin resistance: a three-armed randomized controlled trial. Hum Reprod. 2022;37:542–52. 10.1093/humrep/deab272. 34907435 10.1093/humrep/deab272 PMC8888993

[30] Liang F, Koya D. Acupuncture: is it effective for treatment of insulin resistance? Diabetes Obes Metab. 2010;12:555–69. 10.1111/j.1463-1326.2009.01192.x. 20590731 10.1111/j.1463-1326.2009.01192.x

[31] Firouzjaei A, Li GC, Wang N, Liu WX, Zhu BM. Comparative evaluation of the therapeutic effect of metformin monotherapy with metformin and acupuncture combined therapy on weight loss and insulin sensitivity in diabetic patients. Nutr Diabetes. 2016;6:e209. 10.1038/nutd.2016.16. 27136447 10.1038/nutd.2016.16 PMC4895377

[32] Li L, Zhang J, Zeng J, Liao B, Peng X, Li T, et al. Proteomics analysis of potential serum biomarkers for insulin resistance in patients with polycystic ovary syndrome. Int J Mol Med. 2020;45:1409–16. 10.3892/ijmm.2020.4522. 32323743 10.3892/ijmm.2020.4522 PMC7138261

[33] Benrick A, Kokosar M, Hu M, Larsson M, Maliqueo M, Marcondes RR, et al. Autonomic nervous system activation mediates the increase in whole-body glucose uptake in response to electroacupuncture. FASEB J. 2017;31:3288–97. 10.1096/fj.201601381R. 28404742 10.1096/fj.201601381R

[34] Duan L, An X, Zhang Y, Jin D, Zhao S, Zhou R, et al. Gut microbiota as the critical correlation of polycystic ovary syndrome and type 2 diabetes mellitus. Biomed Pharmacother. 2021;142:112094. 10.1016/j.biopha.2021.112094. 34449321 10.1016/j.biopha.2021.112094

[35] Yan M, Man S, Sun B, Ma L, Guo L, Huang L, et al. Gut liver brain axis in diseases: the implications for therapeutic interventions. Signal Transduct Target Ther. 2023;8:443. 10.1038/s41392-023-01673-4. 38057297 10.1038/s41392-023-01673-4 PMC10700720

[36] Sayin SI, Wahlstrom A, Felin J, Jantti S, Marschall HU, Bamberg K, et al. Gut microbiota regulates bile acid metabolism by reducing the levels of tauro-beta-muricholic acid, a naturally occurring FXR antagonist. Cell Metab. 2013;17:225–35. 10.1016/j.cmet.2013.01.003. 23395169 10.1016/j.cmet.2013.01.003

[37] Ji C, Xu W, Zhang Z, Cui S, Yi W. Effect of electroacupuncture on reproductive disorders and insulin resistance in a murine polycystic ovary syndrome model. Evid Based Complement Alternat Med. 2021;2021:9968463. 10.1155/2021/9968463. 34987599 10.1155/2021/9968463 PMC8720607

[38] Lin Y, Zeng H, Lin J, Peng Y, Que X, Wang L, et al. Evaluating the therapeutic potential of moxibustion on polycystic ovary syndrome: a rat model study on gut microbiota and metabolite interaction. Front Cell Infect Microbiol. 2024;14:1328741. 10.3389/fcimb.2024.1328741. 38665877 10.3389/fcimb.2024.1328741 PMC11043641

[39] Huang S, Yu C, Hu M, Wen Q, Wen X, Li S, et al. Electroacupuncture ameliorates hepatic defects in a rat model of polycystic ovary syndrome induced by letrozole and a high-fat diet. Acupunct Med. 2024;42:87–99. 10.1177/09645284231207863. 38044823 10.1177/09645284231207863

[40] Li D, Bai P, Wu JY, Xie M, Zhao RZ, Wang ZP, et al. Effect of acupuncture on ovary morphology and function in DHEA-induced polycystic ovary syndrome model rats. Chin J Integr Med. 2021;27:220–4. 10.1007/s11655-021-3290-0. 33666871 10.1007/s11655-021-3290-0

[41] Watanabe M, Houten SM, Mataki C, Christoffolete MA, Kim BW, Sato H, et al. Bile acids induce energy expenditure by promoting intracellular thyroid hormone activation. Nature. 2006;439:484–9. 10.1038/nature04330. 16400329 10.1038/nature04330

[42] Zhang W, Wu F. Elevated linoleic acid intake becomes a risk factor for polycystic ovary syndrome by affecting ovarian granulosa cells. FASEB J. 2025;39:e70518. 10.1096/fj.202402648RR. 40197608 10.1096/fj.202402648RR PMC11977604

[43] Selen ES, Bolandnazar Z, Tonelli M, Butz DE, Haviland JA, Porter WP, et al. NMR metabolomics show evidence for mitochondrial oxidative stress in a mouse model of polycystic ovary syndrome. J Proteome Res. 2015;14:3284–91. 10.1021/acs.jproteome.5b00307. 26076986 10.1021/acs.jproteome.5b00307 PMC6681913

[44] Zou Y, Zhu FF, Fang CY, Xiong XY, Li HY. Identification of potential biomarkers for urine metabolomics of polycystic ovary syndrome based on gas chromatography-mass spectrometry. Chin Med J (Engl). 2018;131:945–9. 10.4103/0366-6999.229899. 29664055 10.4103/0366-6999.229899 PMC5912061

[45] Hu R, Geng Y, Huang Y, Liu Z, Li F, Song K, et al. Jiawei buzhong yiqi decoction attenuates polycystic ovary syndrome through regulating kisspeptin-GPR54-AKT-SHBG system. Phytomedicine. 2024;133:155931. 10.1016/j.phymed.2024.155931. 39116604 10.1016/j.phymed.2024.155931

